# Tolerance of immersive head-mounted virtual reality among older nursing home residents

**DOI:** 10.3389/fpubh.2023.1163484

**Published:** 2023-07-19

**Authors:** Hajer Rmadi, Pauline Maillot, Romain Artico, Edouard Baudouin, Sylvain Hanneton, Gilles Dietrich, Emmanuelle Duron

**Affiliations:** ^1^Institut des Sciences du Sport-Santé de Paris (URP3625 – I3SP), Université Paris Cité, Paris, France; ^2^Laboratoire Complexité, Innovations, Activités Motrices et Sportives (CIAMS, EA4532), Université Paris-Saclay, Orsay, France; ^3^Inserm, CESP, Team MOODS, Université Paris-Saclay, Le Kremlin-Bicêtre, France; ^4^Assistance Publique - Hôpitaux de Paris, Department of Geriatric, Paul Brousse Hospital, Villejuif, France

**Keywords:** older adults, virtual reality, tolerance, cybersickness, anxiety

## Abstract

**Introduction:**

Virtual Reality (VR) is a tool that is increasingly used in the aging population. Head-Mounted Displays (HMDs) are stereoscopic vision devices used for immersive VR. Cybersickness is sometimes reported after head-mounted display (HMD) VR exposure. Cybersickness severity and anxiety state reflect VR low tolerance. We aimed to evaluate HMD VR tolerance among older nursing home residents through cybersickness and anxiety state.

**Methods:**

A total of 36 participants were included in this preliminary study, 33 of whom (mean age: 89.33 ± 5.48) underwent three individual HMD VR sessions with three different contents. Cybersickness occurrence and severity were scored by the Simulator Sickness Questionnaire (SSQ) after each session. Anxiety state was assessed by the State–Trait Anxiety Inventory form Y-A before and after each session. Anxiety trait (using State–Trait Anxiety Inventory form Y-B) was also evaluated before and after the experiment. In total, 92% (33/36) of patients completed all three sessions, of which 61% (20/33) did not report any cybersickness symptoms (SSQ = 0). Six participants reported significant cybersickness (defined by an SSQ score ⩾10) in at least one session.

**Discussion:**

Only two participants stopped the study after the first exposure because of cybersickness. Age, cognitive function, anxiety trait, and well-being were not associated with cybersickness. The mean anxiety state decreased significantly from pre- to post-session. This immersive HMD VR experience was well tolerated among nursing home dwellers. Further larger studies in this population aiming to identify CS determinants are needed in order to use HMD VR on a standard basis.

## 1. Introduction

Populations around the world are aging (1), and most older adults suffer from at least one chronic condition (2). Chronic diseases lead to loss of autonomy (3), lower quality of life (4), and institutionalization (5).

Thus, innovative interventions are needed to promote a better quality of life. Virtual Reality (VR) is growing in the gerontology field (6). VR uses computer science (hardware and software) to simulate virtual environments (7). Compared to traditional displays, such as computer monitors, Head-Mounted Displays (HMDs) isolate the user from the surrounding environment, offer stereoscopic vision, and adjust visual information to the user’s head movements. HMDs mainly cover two major senses (sight and hearing), increasing the user’s immersion (8). VR software can greatly vary in design and content (8). The typology of VR content includes 360° videos or photographs, which typically offer high realism. 3D-modeled environments may have varying levels of detail, from highly realistic to minimalist.

VR offers several advantages for healthcare, such as realistic perceptions and reactions, which optimize the patient’s performance (9), motivation, and adherence to rehabilitation (10). On the other hand, Cybersickness (CS) can occur in 60 to 95% of users following exposure to VR with HMDs (11). CS is VR’s most frequent side effect. Symptoms include nausea, vomiting, dizziness, vertigo, headache, loss of concentration, increased fatigue, and in extreme cases, complete incapacitation (12, 13). CS varies from one interface to another. CS in HMD VR tends to be more frequent and severe compared with other types of VR interfaces (i.e., large screen, CAVES, etc.) (14). In fact, it was found that motion sickness symptomology correlated (at post-test) with self-reported claustrophobic anxiety, probably due to the “imprisonment” of the head (15, 16). CS is significantly associated with anxiety state during exposure, which compromises well-being and leads to intolerance (17–19). Anxiety trait, a general tendency to be more anxious in various situations, is the most often investigated personality trait in relation to CS (20). Older adults’ tolerance of VR varies substantially, depending on the content and the subject (see (8) for a review), thus impacting the user’s experience and limiting VR application (21).

Despite its numerous uses in geriatric patients [cognitive training (22), physical rehabilitation (23), and mental health and affective disorders (24)], few studies have investigated VR tolerance in institutionalized adults. Considering the high risk of CS in a frail population, evaluation was found to be of interest in order to ensure the safety of the use of VR technology. Thus, the aim of this preliminary study was to evaluate (i) an immersive HMD VR program’s tolerance, taking into account CS and anxiety, and (ii) its effect on the well-being of dependent nursing home residents as well as their reported experience.

## 2. Materials and methods

### 2.1. Participants and variables

In this interventional multicentric study, 36 participants were recruited from eight nursing homes in Paris, France from July 2021 to January 2022. Inclusion criteria were 75 years of age or older and a Mini-Mental State Examination (MMSE) (25) score of ≥20/30 (the higher the score, the better the cognitive function). To assess this cognitive state, a consensual French version of the MMSE (26) was used. Non-inclusion criteria were major visual impairment, history of epilepsy, orthostatic hypotension within the previous 3 months, psychiatric disorders (Schizophrenia, dissociative disorders, borderline states, paranoia), vestibular or cerebellar syndromes, and the following medications: neuroleptics, tricyclic antidepressants, and antiparkinsonian drugs.

### 2.2. Procedure

#### 2.2.1. Ethics statement

Each participant provided written informed consent for the procedure. The study was approved by the French Ethics Committee East II (2020-A00377-32). The protocol was registered on ClinicalTrials.gov under the reference NCT04365829.

#### 2.2.2. Participants

Participants’ age, MMSE, and gender were collected. A total of 33 older adults were included out of the 36 who were screened. The mean age was 89.33 years old ±5.48, and 72.7% (n = 24) were women. The men’s age was (mean ± standard deviation) 87 ± 5.17 and they had an MMSE score of 26.22 ± 2.91. The women’s age was 90.21 ± 5.34 and they had an MMSE score of 25.13 ± 3.14.

**Figure 1:**
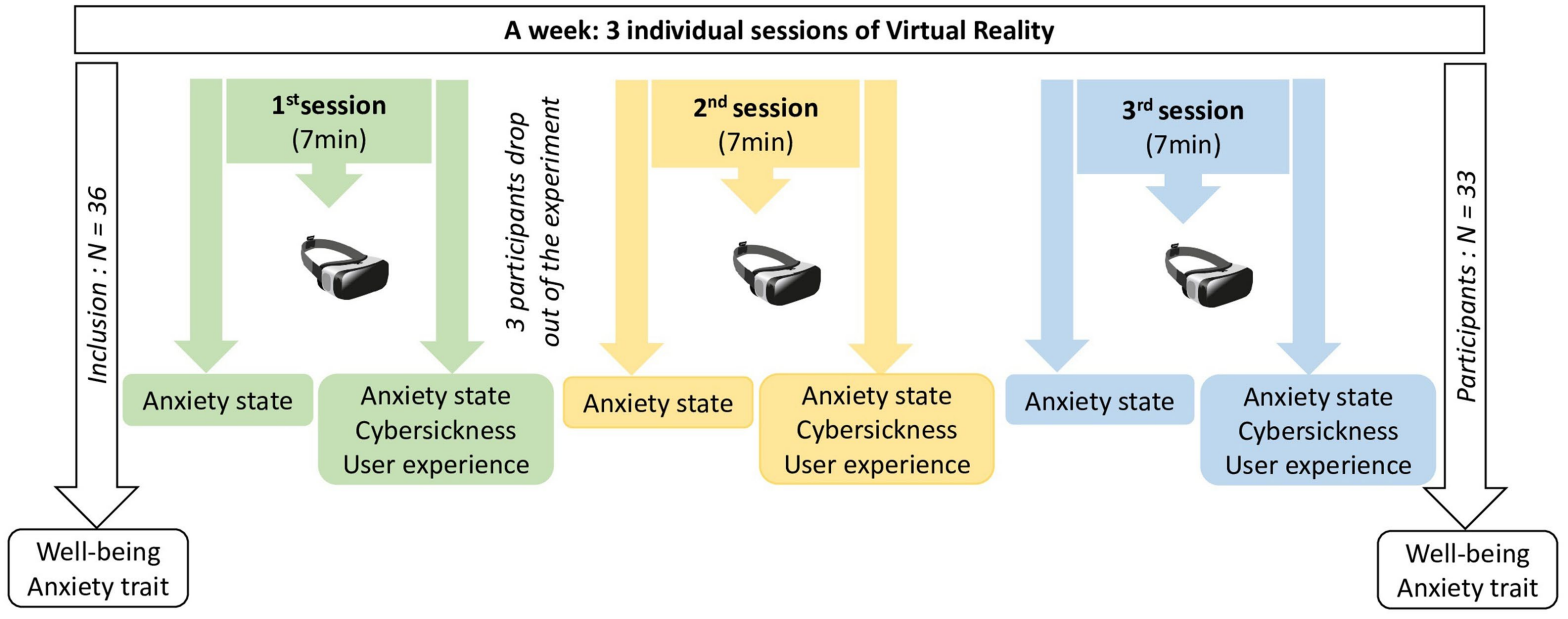
Schematic representation of the protocol including the order of evaluations.

One participant dropped out after the first session due to a lack of motivation. Two participants (mean age 88.5 ± 2.12 and mean MMSE 29 ± 1.41) refused further participation during the first session because of VR intolerance (Figure 1).

#### 2.2.3. HMD VR exposure

Each participant underwent three individual VR sessions (S1, S2, S3) within a week (Figure 1). Each session (7-min exposure) took place individually in a quiet room. VR sessions were conducted using the Lumeen^1^ software, which allowed the session to be controlled and monitored with a digital tablet, installed on a Huawei^2^ Mediapad T5 tablet and a Pico G2 4K^3^ HMD.

**Figure 2:**
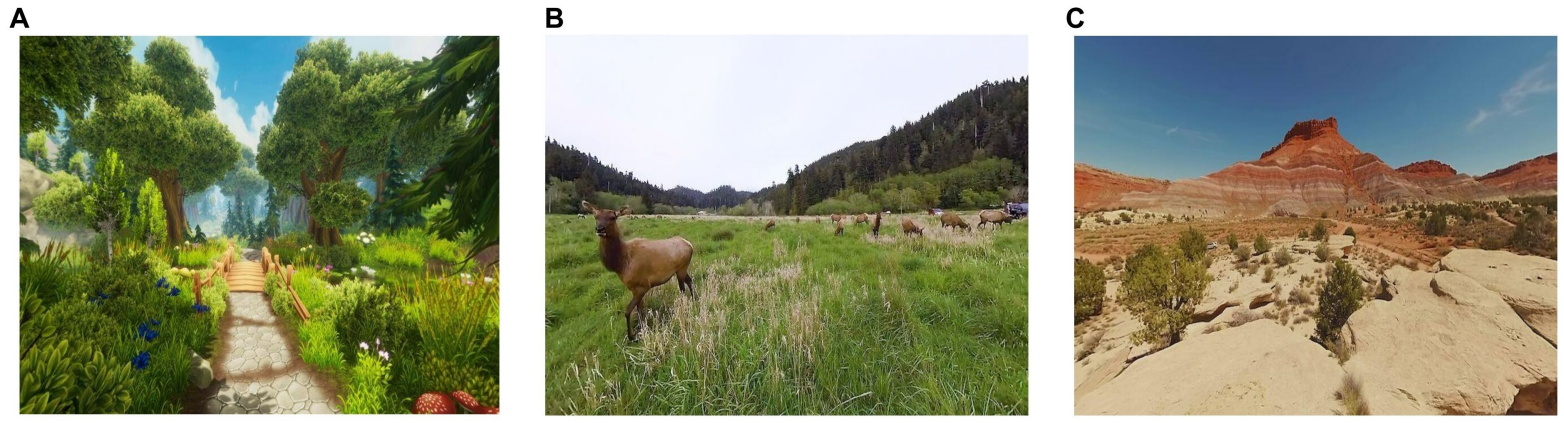
Prereviews from the Forest Through the Seasons scene **(A)**, Animals of the World scene **(B)**, and The Grand Canyon scene **(C)**.

The immersive experiences consisted of 7-min 360° videos selected from Lumen’s catalog. Participants watched only one of the following VR scenes per session: (a) Forest Through the Seasons, a 360° animation film (pre-rendered 3D graphics) in a cartoon style showing the changes of a forest landscape, (b) Animals of the World, a 360° live-action film of animals in the wild, and (c) The Grand Canyon, a 360° live-action film of a visit to the Grand Canyon (Figure 2). The virtual environment projected in the headset was coupled to the participant’s head movement. They had no monitoring tool allowing them to start or stop the VR environment and no other possible interaction. The scene sequence was randomized by drawing at each session.

#### 2.2.4. Outcomes

VR tolerance was studied using cybersickness (CS) occurrence and anxiety questionnaires.

Cybersickness was measured by the Simulator Sickness Questionnaire (SSQ) (12). This questionnaire has been previously used in several studies to assess cybersickness (27, 28). The French-Canadian translation (29) of this questionnaire, measuring VR’s side effects among a sample of French-speaking participants, was used. Each of the 16 SSQ items are rated on a 4-point scale: 0 = none, 1 = slight, 2 = moderate, and 3 = severe. They were quoted by the investigator according to answers given by the participants.

Sub-scores of nausea, oculomotor disorder, and disorientation were calculated, and the global score is the weighted average of these sub-scores (12). According to the categorization of Stanney et al. (30), symptoms are considered “negligible” when the global score is <5, “minimal” when between 5 and 10, “significant” when between 10 and 15, and “concerning” when comprised between 15 and 20. Tolerance is considered low when the score is above 20. The population was divided into two groups: SSQ ⩾ 10 at least once vs. < 10 in all three sessions.

Anxiety was assessed by the State–Trait Anxiety Inventory (STAI) (31). The French version (IASTA-Y65+) of this questionnaire, adapted and validated for older adults aged between 65 and 92 (M = 77.5, SD = 7), was used (32). It is composed of two forms (Y-A and Y-B). The two forms of this questionnaire have excellent reliability with a Cronbach’s alpha coefficient, respectively = 0.91 and 0.93; *p* < 0.05. Furthermore, a high test–retest stability was found for the two forms of the STAI (*p* < 0.001) (32). Y-A was used to assess the participant’s anxiety state (which refers to anxiety in a specific moment) before and after each VR exposure. Y-B was used before the first and after the third session to assess participant anxiety trait (which refers to anxiety as a trait of personality). Each form scored from 20 to 80; the higher the score, the more anxious the patient.

Well-being was assessed using the Well-Being Index (WHO-5) questionnaire before the first and after the third session (33). The French version published by the (34), validated among older people aged 70.2 years ±8.0, was used (33). This questionnaire has been shown to have good internal consistency and homogeneity among this population (33). Furthermore, recently, the WHO-5 was found to be reliable in 35 countries (including France) and has parameters that do not vary across countries (35). It is composed of five questions (from 0 to 5 points; the higher the score, the better the well-being) (5: All the time, 4: Most of the time, 3: More than, 2: Less than half the time half of the time, 1: From time to time, 0: Never). The total score ranges from 0 to 100.

User experience was assessed after each session using a questionnaire designed specifically for the study. Participants responded to one item assessing perceived usefulness (“Overall, I find this tool interesting”), two assessing perceived enjoyment (e.g., I find the experience relaxing), two assessing perceived ease of use (e.g., “I find the device comfortable”), one assessing intention to use (“I would like to use this device again”) on a Likert scale from 1 (strongly disagree) to 6 (strongly agree). These items were developed based on items commonly used in the Technology Acceptance Model (36) and on other studies specifically investigating technology acceptance by older adults (37–39). The total score ranges from 6 to 36. The higher the score, the greater the experience reported by the participant.

#### 2.2.5. Statistics

Statistical analyses were performed with the JASP 0.16.0 software (which uses R as back-end). Normality was assessed using a Shapiro–Wilk test. The significance of the results was retained for a value of p less than 0.05. Cohen’s d was used to characterize the effect size.

The sample size was calculated to ensure a significant difference (5% significance level) between the SSQ scores of CS and a theoretical score of 20 (the score at which CS is severe) (30). For an expected medium effect size of 80% power, the required sample size was 27.

Inference statistics were done by Student t-test for independent samples, ANOVA repeated measures tests were used on SSQ, STAI form Y-A results, and acceptance questionnaire scores. The Holm’s *post hoc* test was used on STAI form Y-A scores. A paired t-test was used on STAI form Y-B scores before and after the experiment and on WHO-5 scores.

Descripted statistics were used to qualify the participant’s experience.

## 3. Results

### 3.1. Cybersickness

The global SSQ (mean ± standard deviation) was 1.84 ± 3.44. Nausea sub-scores were 0.48 ± 2.72, oculomotor disorder was 2.29 ± 4, and disorientation was 1.96 ± 4.71. Subgroup analysis is provided in Table 1. SSQ scores did not vary significantly over the sessions [*F* (2, 32) = 1.22; *p* = n.s.] or over the contents [*F* (2,31) = 0.005; *p* = n.s.]. The mean SSQ score for the two participants who left the experiment because of VR intolerance after the first session was 63.56 ± 10.57. CS evaluation among participants who completed the three sessions showed that HDM VR exposure caused important symptoms, with an SSQ score of ⩾ 10 in 8% of cases (8/99 sessions completed). There was no effect of gender on SSQ score (*p* = n.s). Six participants (18%) had an SSQ of ⩾10 at least during one session (mean SSQ 7.89 ± 4.14); of these, five (83%) were women. Among the participants, 61% (20/33) did not report any CS symptoms in any of the sessions and 21% (7/33) had signs of CS during at least one session (mean SSQ 1.95 ± 0.98).

**Table 1:** Characteristics comparison of participants with SSQ score ⩾10 during at least one session vs. SSQ score < 10 during all three sessions.

	SSQ < 10 N = 27		SSQ⩾10 N = 6		*t*	*p*	Cohen’s *d*
	*M*	*SD*	*M*	*SD*			
Age	88.89	5.63	91.33	4.63	−0.99	0.33	−0.45
MMSE	25.52	3.04	25	3.46	0.37	0.71	0.17
AT before	32.63	7.51	34.17	5.91	−0.47	0.64	−0.21
WB before	58.37	25.30	49.33	27.33	0.78	0.44	0.35

M, Mean, SD, Standard Deviation, AT, Anxiety Trait, WB, Well-Being.

Age, MMSE, anxiety trait, and well-being did not vary significantly between the participants who suffered from cybersickness at least once in the three sessions (SSQ score ⩾10) and those who had an SSQ score of <10 (Table 1). There was no effect of repetition [*F* (2, 32) = 1.22; *p* = n.s] and no effect of VR contents [*F* (2,31) = 0.005; *p* = n.s] on CS occurrence.

### 3.2. Anxiety

**Figure 3:**
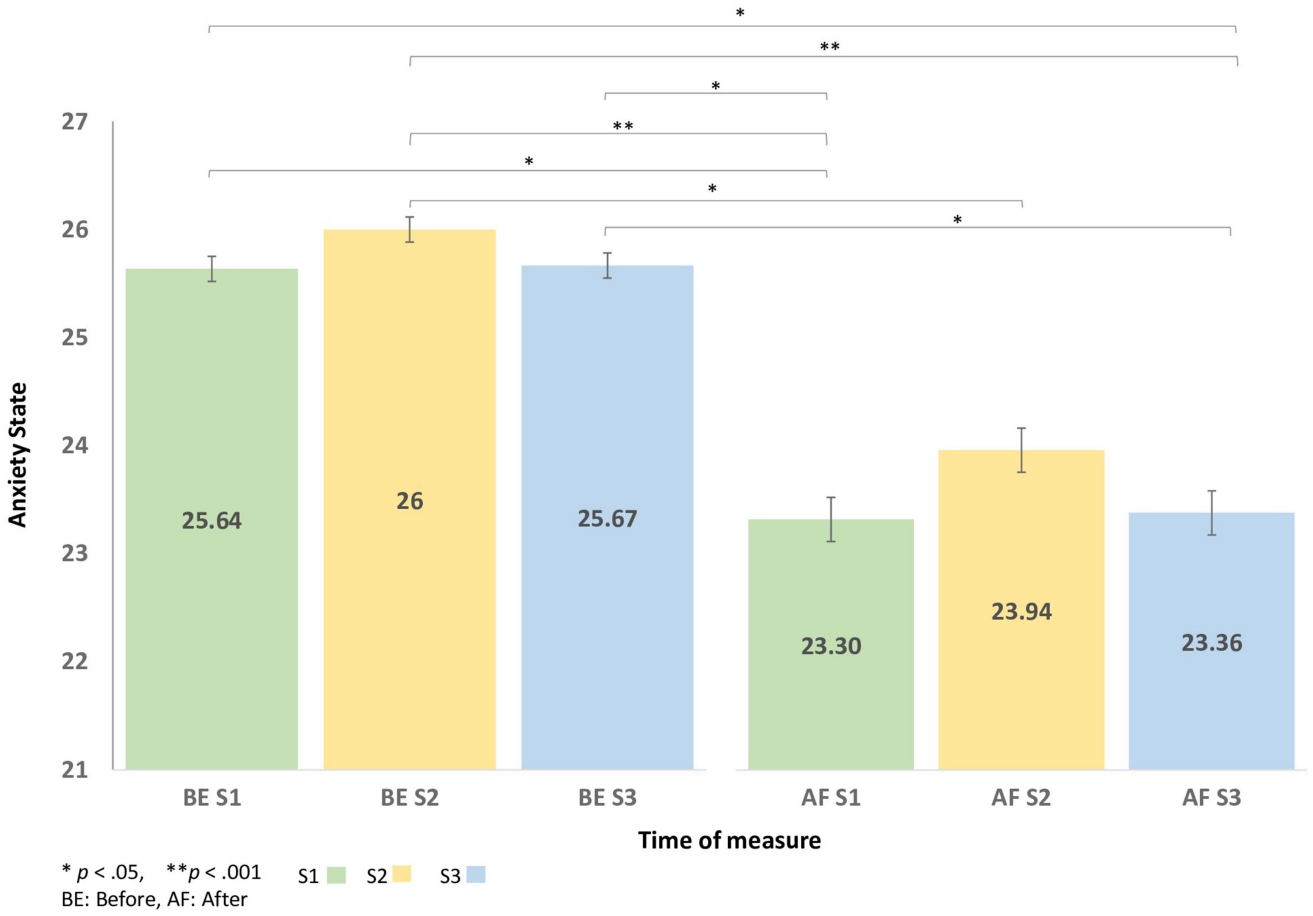
The change in State–Trait Anxiety Inventory (STAI) from Y-A scores before and after each virtual reality session.

Significant differences *F* (32, 160) = 6.12*; p* < 0.001, η^2^ = 0.16 in the STAI from Y-A scores were found between measurements. The Holm’s *post hoc* test revealed that the score decreased significantly after S1 (*p* < 0.05), S2 (*p* < 0.05), and S3 (*p* < 0.05). It increased significantly before S2 (*p* < 0.01) then before S3 (*p* = n.s). Anxiety state decreased significantly after each session (Figure 3). No significant difference between the baseline and the final assessment of participant anxiety trait was observed [t (32) = 0.66; *p* = n.s.].

### 3.3. Well-being

VR sessions’ effect on well-being was not significantly different before (mean 56.73 ± 25.48) and after the experiment (mean 60.61 ± 22.61) [t (32) = −1.13; *p* = n.s.]. The experiment had no significant effect on participants’ subjective psychological well-being.

### 3.4. User experience

The global user experience score was (mean 29.92 ± 4.53) for the HMD three-session VR program. The mean scores and standard deviation for each variable of the questionnaire are represented in Table 2.

**Table 2:** User’s experience results for each variable of the questionnaire.

Variables	Items	*M*	*SD*
Perceived usefulness	Overall, I find this tool interesting	4.37	1.29
Perceived enjoyment	I find the experience relaxing I find the experience boring	5.20	0.75
Perceived ease of use	I find the device comfortable I find the experience exhausting	5.44	0.80
Intention to use	I would like to use this device again	4.31	1.38

M, Mean, SD, Standard deviation.

## 4. Discussion

This study shows that three immersive VR sessions with HMD tend to be tolerated among nursing home residents, whereas no significant effect on well-being was found.

A total of 61% (20/33) of participants did not report any CS symptoms during the study. This is in agreement with a previous study (40), where no participants (mean age 74.8 years old ±10.4) reported severe discomfort on the SSQ scale when exposed to natural and familial HMD VR scenes. Two participants left the study because of CS intolerance and 18% (6/33) experienced significant CS symptoms (⩾10) during at least one session. These symptoms were, however, minimal (mean SSQ < 10) and might have been due to the realistic properties of the VR content. Indeed, two of the proposed scenes are 360° realistic live-action films as opposed to the artificially made VR environment experienced in a similar study Huygelier et al. (40). It has already been reported that levels of immersion and realism can influence CS occurrence: the more realistic the VR environment, the higher the CS occurrence (41, 42).

In contrast, some authors have suggested that older adults have a high risk of CS (43, 44) with an SSQ score higher for subjects of 50 years and older than younger subjects (45). The high tolerance in this study may be due to the short time of exposure (7 min), as shorter duration reduces CS (46, 47). Indeed, higher CS rates were found among 118 participants (70 to 90 years old), where sessions lasted 15 min (44). Second, a meta-analysis showed that current-generation VR HMD induces less CS than previous ones (11, 48). Third, VR contents in this study were comprised only of visual interaction with an adaptation to the head movement. This avoids sensorial disparities during the exposure and prevents CS (49).

In order to explain CS susceptibility among the 18% of participants who expressed significant SSQ, we investigated the relationships between participants’ characteristics and CS severity. CS occurrence was not implicated by age, MMSE, anxiety trait, and psychological well-being. These results do not in agreement with a meta-analysis, which reports that age and psychological disorder (suffering from significant phobia) are related to VR sickness. Furthermore, it is still not clear whether cognitive abilities are correlated with CS (8).

In our study, anxiety state was considered a non-tolerance indicator. This is in agreement with a review that reported that anxiety before, during, or after VR exposition was associated with VR sickness severity (20). Thus, the decreased anxiety state after each session is in favor of good tolerance of the immersive HMD VR program. This result is also in agreement with a previous study, where an immersive VR reminiscence program with historic live-action scenes and computer-generated images reduced the anxiety state of older adults [mean age (SD): 87.1 (4.2) and mean MMSE (SD): 28.5 (1.2)/30].

This study has several strengths. First, it was conducted among older nursing home residents, a population rarely studied in this field. Second, three different HMD VR contents were studied. Third, the use of the STAI questionnaire provides immediate post-exposure data. Fourth, the questionnaire reflects the user’s experience, which is infrequently researched among this population. However, we acknowledge some limitations. First, medical conditions were not recorded. Second, anxiety state was low, which leads us to extrapolate our results to a more anxious population. Third, the proposed VR experience was certainly immersive but not truly interactive, not allowing a comprehensive assessment of VR tolerance. Finally, to assess user experience, we used a non-validated questionnaire designed to answer specific questions related to VR acceptance. This questionnaire could produce biased results by changing the participants’ self-awareness.

## 5. Conclusion

To conclude, an immersive HMD VR experience was well tolerated among dependent nursing home residents. VR exposure also transiently reduced anxiety without important side effects (except some light CS symptoms) in most participants. Given the good tolerance, it would be interesting to evaluate the effectiveness of a VR program. Further larger studies in this population aiming to identify CS determinants are needed in order to use HMD VR on a standard basis. Finally, it would be useful to take into account the caregiver’s opinion in a prospective study with VR exposure.

## Data availability statement

The original contributions presented in the study are included in the article/supplementary material, further inquiries can be directed to the corresponding author.

## Ethics statement

The studies involving human participants were reviewed and approved by the French Ethics Committee East II (2020-A00377-32). The patients/participants provided their written informed consent to participate in this study.

## Author contributions

All authors listed have made a substantial, direct, and intellectual contribution to the work and approved it for publication.

## Funding

This work was supported by Ile de France Region.

## Conflict of interest

The authors declare that the research was conducted in the absence of any commercial or financial relationships that could be construed as a potential conflict of interest.

## Publisher’s note

All claims expressed in this article are solely those of the authors and do not necessarily represent those of their affiliated organizations, or those of the publisher, the editors and the reviewers. Any product that may be evaluated in this article, or claim that may be made by its manufacturer, is not guaranteed or endorsed by the publisher.

## Footnotes

^1^Lumeen, 213 rue de Gerland, 69,007 Lyon, France, https://www.lumeen.com/.

^2^Huawei Technologies Co., Ltd. Bantian, Longgang District, Shenzhen, 518,129, P.R.C. https://www.huawei.com/.

^3^Pico Immersive Pte. Ltd., 1 Raffles Quay #26–10, Singopore (048583), https://www.picoxr.com/.
